# Challenges to mapping the health risk of hepatitis A virus infection

**DOI:** 10.1186/1476-072X-10-57

**Published:** 2011-10-18

**Authors:** Khayriyyah Mohd Hanafiah, Kathryn H Jacobsen, Steven T Wiersma

**Affiliations:** 1 Expanded Program on Immunization, Department of Immunization, Vaccines, and Biologicals, World Health Organization, 20 Avenue Appia, 1211 Geneva 27, Switzerland; 2 Department of Global & Community Health, George Mason University, 4400 University Drive MS 5B7, Fairfax, Virginia 22030, USA

**Keywords:** hepatitis A, geographic information systems, health risk maps, risk mapping, vaccine recommendations, global health, travel health

## Abstract

**Background:**

World maps are among the most effective ways to convey public health messages such as recommended vaccinations, but creating a useful and valid map requires careful deliberation. The changing epidemiology of hepatitis A virus (HAV) in many world regions heightens the need for up-to-date risk maps. HAV infection is usually asymptomatic in children, so low-income areas with high incidence rates usually have a low burden of disease. In higher-income areas, many adults remain susceptible to the virus and, if infected, often experience severe disease.

**Results:**

Several challenges associated with presenting hepatitis A risk using maps were identified, including the need to decide whether prior infection or continued susceptibility more aptly indicates risk, whether to display incidence or prevalence, how to distinguish between different levels of risk, how to display changes in risk over time, how to present complex information to target audiences, and how to handle missing or obsolete data.

**Conclusion:**

For future maps to be comparable across place and time, we propose the use of the age at midpoint of population susceptibility as a standard indicator for the level of hepatitis A endemicity within a world region. We also call for the creation of an accessible active database for population-based age-specific HAV seroprevalence and incidence studies. Health risk maps for other conditions with rapidly changing epidemiology would benefit from similar strategies.

## Background

Maps are valuable tools for epidemiologic research and application, and are particularly effective in communicating key public health messages to a wide range of audiences. One example of the importance of global risk maps relates to the display of vaccination recommendations for hepatitis A virus (HAV), an infectious disease strongly linked to income, access to clean water, and access to sanitation [1]. Most children in low-income areas become infected in early childhood when HAV infections are typically asymptomatic, and infection confers lifelong immunity. Thus, in low-income areas the incidence of infection is usually high, but the disease burden is low and hepatitis A is not considered to be a major public health problem. In contrast, hepatitis A is a growing public health concern in high-income areas where the infection rate is usually low, since many adults remain susceptible to HAV and are at risk of severe symptoms and death [2-4]. Additionally, hepatitis A may cause a significant economic burden to individuals, families, and communities, especially in areas with a sizeable proportion of susceptible older adults [5-7]. This is the paradox of hepatitis A risk, and one of the reasons why mapping this risk is such a challenge: residents of areas with a high infection transmission rate have a lower rate of severe disease and death than those living in areas with intermediate or low infection rates.

The most recent global estimates of anti-HAV IgG seroprevalence suggest that an epidemiological transition is occurring in many countries and regions, with the incidence of infection in most of the world declining [8,9]. As a result, the proportion of susceptible adults in these countries is increasing. Contaminated food and water are responsible for a considerable proportion of incident HAV cases, and the globalization of the food system, which increases the likelihood of food from endemic areas being shipped to and consumed in low-endemicity areas, may place susceptible adults and children in low anti-HAV prevalence areas at risk [10-12]. Travel from low-endemicity areas to places where hepatitis A is endemic is also often reported as a source of infection [13-17]. Just as with infection acquired from imported foods, returning travelers may bring the infection to their hometowns and spark community outbreaks [2,6,13,15,17-19].

Because of these changes in HAV risk and, consequently, the shifting epidemiologic profiles of HAV in diverse parts of the world, there is a pressing need for up-to-date information and risk maps for both residents of areas in transition and travelers to these areas. This information is required by public health authorities making immunization policies, health care practitioners making recommendations about vaccination, and individuals making health decisions for themselves and their families. Maps are one of the most effective ways to rapidly convey information about global issues [20]. However, maps can only display a limited amount of information, and complex decisions must be made about what information to display and how best to display it. This paper examines and compares the features of several commonly used hepatitis A risk maps, characterizes the key challenges of displaying hepatitis A risk on a map based on observations of these widely-used maps, and outlines potential approaches to improving maps for use by policymakers and health practitioners.

## Results

Based on our comparison of five commonly-referenced hepatitis A risk maps (Table 1), we identified five key challenges associated with presenting hepatitis A risk:

**Table 1:** Comparison of commonly accessed hepatitis A maps.

Map Source	Title	Data Sources Listed	Target Audience	Risk Categories	Cutoffs for Risk Categories Listed	Notes
CDC: Travelers' Health 2008 (Yellow Book) [24]	"Prevalence of antibody to hepatitis A virus, 2006"	No	Travel clinics and travelers from low prevalence areas	Prevalence of antibody to Hepatitis A Virus: * High * Intermediate * Low	No	"Estimates of prevalence of antibody to hepatitis A virus (anti-HAV), a marker of previous HAV infection, are based on limited data and might not reflect current prevalence. In addition, anti-HAV prevalence might vary within countries by subpopulation and locality. As used on this map, the terms 'high,' 'medium,' and 'low' endemicity reflect available evidence of how widespread HAV infection is within each country, rather than precise quantitative assessments."

WHO: International Travel & Health Report (2008) [23]	"Hepatitis A, countries or areas at risk"	No	Travel clinics and travelers from low prevalence areas	* Countries or areas with moderate to high risk	No	"The risk of infection is based on estimated prevalence rate of antibody to hepatitis A (anti-HAV) - a marker of previous HAV infection - among population. This marker is based on limited data and may not reflect current prevalence."

Jacobsen, 2010 [9]	"Estimated prevalence of hepatitis A virus, 2005"	Yes	Clinicians and researchers	Anti-HAV endemicity: * High * Intermediate * Low * Very low	Yes, by percent immune by age	"Evidentiary support indicated as very limited, limited, moderate, or extensive based on number of articles per country in each world region."

Nothdurft, 2008 [26]	"Worldwide pattern of the prevalence of hepatitis A virus"	Yes	Clinicians and researchers	Anti-HAV prevalence: * High * Transitional * Intermediate * Low or Very Low	No	None listed.

Bell, 2002 [25]	"Geographic distribution of the prevalence of hepatitis A virus"	No	Clinicians and researchers	Anti-HAV prevalence: * High * Transitional * Intermediate * Low or Very Low	No	"Based on summary of available data."

### 1. Defining high risk

**Figure 1:**
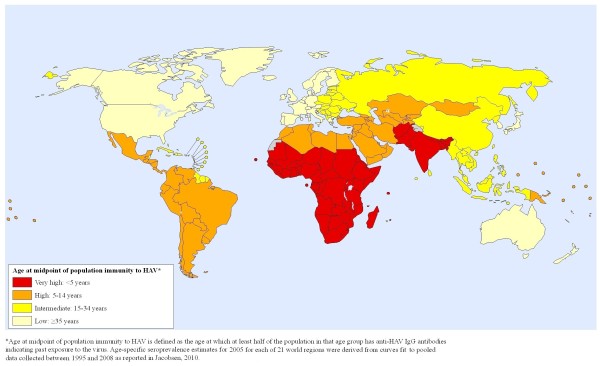
**Global risk map of HAV immunity in 2005**.

Most maps can only display one aspect of risk, and this is a challenge for hepatitis A since there are multiple ways to define and present risk. All of the five maps we examined used some indicator of the seroprevalence rate (the proportion of a population with immunity to hepatitis A based on past infection), often reported as the more generic "endemicity" rather than as specific percentages, as their measure of risk. A high seroprevalence rate, especially a high rate among children, is evidence of a high incidence rate (the rate of new infection) in the population. Individuals living in areas with high incidence rates usually acquire the infection in early childhood, when the risk of severe disease is minimal. Maps that illustrate anti-HAV immunity rates across the globe are useful for persons traveling from areas with low incidence rates to areas with high transmission rates since these individuals may have a significant risk of becoming infected during travel if they have not had prior infection or immunization (Figure 1).

**Figure 2:**
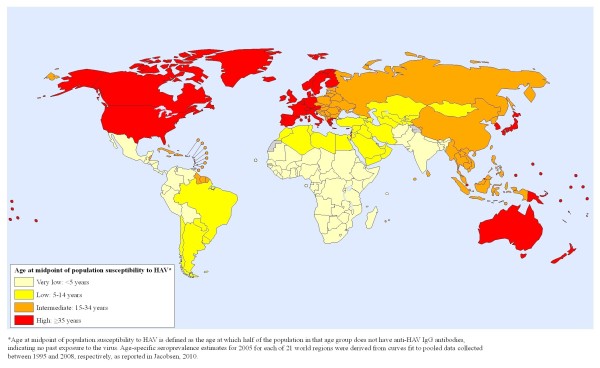
**Global risk map of HAV susceptibility in 2005**.

However, the opposite scale--one designating areas with a high proportion of *susceptible *adults as the "high risk" category on the map rather than showing *immunity *as the evidence of high risk--might better convey the risk of severe disease. A map showing adult susceptibility (Figure 2) might more effectively indicate where the case fatality rate is likely to be highest should an outbreak occur, since the risk of severe morbidity and mortality increases with increasing age. This message about risk might be the most appropriate one for policymakers from regions with moderate or high susceptibility rates, since these may be the areas where hepatitis A has the greatest likelihood of becoming a major public health problem. Additionally, such a map would warn travelers from "high risk of susceptibility" areas that they are likely to be at risk of hepatitis A disease and should be vaccinated before international travel to regions of the world with different hepatitis A profiles.

One limitation of using seroprevalence data rather than incidence data is that the majority of adults in a population with immunity to HAV probably acquired the infection decades ago when they were young children, so prevalence data may capture past conditions much more than current risks. Thus, a risk map might best display incidence rates rather than seroprevalence rates. However, HAV infection incidence is often very difficult to measure. Case detection and reporting are very low since many cases of HAV infection are asymptomatic or have only mild symptoms, especially among children. Furthermore, the capacity for conducting hepatitis A surveillance varies by country.

### 2. Defining levels of risk

Defining the values that separate categories of risk is another important consideration for mapmakers. Since there are no well-established definitions for which incidence or prevalence rates constitute high or low hepatitis A risk, most of the five hepatitis A maps we examined present vague categories such as "endemicity level" rather than providing a key showing the quantitative criteria used to assign each entity on the map to one of the risk categories. One outcome of the different, and usually undisclosed, scales used by different mapmakers is that the resulting maps lack comparability. However, attempting to be more precise about the rates displayed might be inappropriate for two reasons.

The first concern is that many countries lack current data about hepatitis A incidence and anti-HAV seroprevalence rates. In the absence of good data, mapmakers must make "best guesses" about the likely transmission rate in an area. This requires making judgment calls about whether to assume that the risk profile has not changed (likely remaining "high") or to assume that over time the risk profile has changed (maybe becoming "moderate"), perhaps based on a comparison of relatively nearby places with more current data. Assigning these estimates to categories listing actual incidence or prevalence rates may imply a greater level of certainty about the classification than is appropriate. Maps created without good primary data may be interpreted to be authoritative by policymakers and others who are involved in making important decisions about vaccination recommendations, public health initiatives, and other potentially costly interventions, even if the mapmakers are candid about data limitations.

The second concern is that even if current data were available from all countries, it might be misleading to assign two regions with very similar seroprevalence rates to different risk categories simply because those rates happen to fall on opposite sides of a cutoff point. When appropriate, the cutoff points should be adjusted to avoid fine distinctions that do not represent differences that are meaningful.

### 3. Capturing changes over time

**Figure 3:**
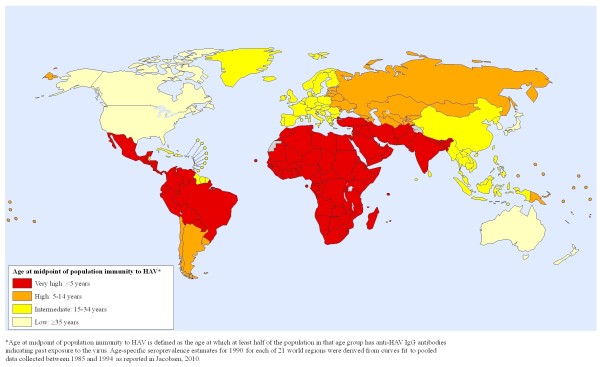
**Global risk map of HAV immunity in 1990**.

Another issue related to the definition of risk categories is whether risk should be defined in relative terms or if cutoffs for higher and lower risk levels should be based on a consistent scale. Significant shifts in hepatitis A risk and susceptibility are occurring in most world regions [8,9], and it is important for risk maps to reflect those changes. For example, Figures 1 and 3 use the same risk definition and cutoffs for anti-HAV seroprevalence to illustrate that some regions transitioned from a high risk category in 1990 (Figure 3) to a lower risk category in 2005 (Figure 1).

If absolute rates are used to assign risk levels, several world regions would move into a lower risk category between 1990 and 2005. If relative rather than absolute measures are used, a world region classified as high risk for hepatitis A in 1990 that experienced a significant decrease in incidence over the subsequent 15 years might still be classified as high risk in 2005 if other regions experienced similar declines in incidence, because that region would continue to have high risk relative to other regions. Maps that obscure meaningful shifts in disease risk over time may hinder the re-evaluation of vaccination policies and other important public health considerations.

### 4. Anticipating user needs

Maps are usually created to meet the needs of one or more specific "consumers" of health information. Several of the maps selected for comparison were made for travelers (or, more specifically, for travelers from areas with low anti-HAV seroprevalence rates and a correspondingly high proportion of susceptible adults). The risk presented by travel health risk maps is intended to translate into vaccination recommendations for travelers. Maps created to guide immunization policy for local residents would look quite different from those designed to display vaccine recommendations for travelers (Table 2).

**Table 2:** Summary of risk of hepatitis A to travelers and typical vaccine recommendations by Global Burden of Disease (GBD) Study region.

Region	**Risk to travelers from low-risk areas **[9]	Typical Vaccine Recommendation	
		
		**for travelers to region **[23,24]	**for residents of region **[6]
South sub-Saharan Africa	Very High	Recommended	Not recommended

West sub-Saharan Africa	Very High	Recommended	Not recommended

South Asia	Very High	Recommended	Not recommended

Central sub-Saharan Africa	Very High	Recommended	Not recommended

East sub-Saharan Africa	Very High	Recommended	Not recommended

Central Latin America	High	Recommended	Targeted

Andean Latin America	High	Recommended	Targeted

Central Asia	High	Recommended	Targeted

Southern Latin America	High	Recommended	Targeted

Tropical Latin America	High	Recommended	Targeted

North Africa/Middle East	High	Recommended	Targeted

Oceania	High	Recommended	Targeted

Caribbean	Intermediate	Recommended	Universal

Southeast Asia	Intermediate	Recommended	Universal

Eastern Europe	Intermediate	Recommended	Universal

East Asia	Intermediate	Recommended	Universal

High-income Asia Pacific	Low	Not recommended	Targeted

Australasia	Low	Not recommended	Targeted

Western Europe	Low	Not recommended	Targeted

High-income North America	Low	Not recommended	Targeted

The process of creating maps that meet the needs of various consumers can involve sensitive decisions about how to portray risk to the target populations. Health and tourism officials in countries with intermediate hepatitis A incidence rates and those from countries with low transmission in cities but high transmission in rural areas may prefer to have their countries presented as low risk areas in order to avoid a negative impact on trade and tourism. Alternatively, health officials may prefer to have their countries displayed as high risk to encourage all potentially susceptible travelers to seek vaccination prior to travel, reducing the risk of travel-related hepatitis A infection and potential secondary spread when the traveler returns home. Similarly, some travel medicine specialists prefer country-level maps that simply state the need for vaccination as present or absent for travelers, while other physicians want to know the risk at a finer spatial scale so they can make more nuanced recommendations to their clients based on their particular travel itineraries.

Additionally, while policymakers are usually looking for hepatitis A maps that display the disease risk for resident *populations*--information that will be used to decide whether universal vaccination, targeted vaccination, or no vaccination appears to be the most appropriate for the population as a whole--travelers and travel medicine specialists want to know the risk to *individual *travelers. No map will ever be fully suitable for an individual traveler, since a traveler's personal vulnerability depends not only on the country of origin and the destination country but also on a host of other factors, such as personal medical history, the duration of the stay, and the activities in which the traveler intends to engage [14]. Even so, health risk maps intended to display risk to populations are often used to make individual health decisions, and mapmakers must reflect on how their maps may be interpreted--and perhaps misinterpreted--and consider how they can most clearly convey the intended use of the map.

### 5. Handling missing or obsolete data

The data sources for most of the maps we examined were not immediately clear, but many countries and regions are known to have incomplete or obsolete data [9]. Furthermore, most world regions have some countries that dominate the available data and some countries for which no recent data are available.

One common way to handle missing data is to map at the regional level rather than using smaller mapping units such as countries. However, this may hide differences known to exist within some regions where ample data are available, and may limit the usefulness of the map [21]. The differences known to exist within some regions may have important policy and practice implications. For example, a susceptible traveler heading to a rural part of an intermediate-risk country may be at significantly increased risk of acquiring hepatitis A compared to a similar traveler who intends to stay in cities. A world map displaying risk that is only accurate to the country level will not provide enough information about heterogeneity within mapped areas to distinguish between the risk profiles of travelers to rural and urban locations.

Another approach is to leave blank those countries or areas lacking current HAV epidemiologic information. However, leaving blank spaces has been shown to interfere with pattern recognition [22], and since most countries without recent data are presumed to have high incidence rates it might be irresponsible not to mark those areas as having high risk for travelers. A third option is to provide an indicator that shows the level of evidentiary support available for each mapping unit, but this may be far too much information to display on a map intended for use by a wide range of consumers.

## Discussion

We identified several inadequacies of current global risk maps for hepatitis A: the definition of risk using vague terms like endemicity rather than incidence or prevalence; the use of inconsistent thresholds for risk categories, which makes it difficult to compare maps and identify trends over time; failure to acknowledge data sources and to identify when data are unavailable or obsolete; and failure to clearly identify appropriate uses of particular maps. These limitations demonstrate the need for improvement in hepatitis A mapmaking practices.

In order for maps to be comparable across place and time, mapmakers need to provide information about the measures of risk they are displaying and the cutoffs they used to distinguish between various risk categories. The inability to accurately measure incidence and uncertainty about how to interpret age-seroprevalence data for HAV have led most mapmakers to instead display a vague "endemicity level" rather than a more concrete measure of risk. One option we support for making "endemicity" a more measurable concept is to adopt the use of "age at midpoint of population susceptibility" as an indicator of endemicity (as illustrated in Figures 1 and 3). The age at midpoint of population susceptibility may be more helpful as an indicator for trends over time than the prevalence at a particular age, even though it would change more slowly than measures that are more immediately affected by incidence rates. Alternatively, the proportion of a population with anti-HAV at age 10 or age 30 or some other age could be an option for defining the endemicity level.

The greatest limitation to making up-to-date global hepatitis A risk maps is the absence of current representative data from many parts of the world. The creation of an active global database of HAV seroprevalence and incidence data by age and jurisdiction would be valuable for risk assessment and for ensuring that new maps are accurate and current. A model for this type of database exists: the European Region of the World Health Organization's Centralized Information System for Infectious Diseases (CISID). This online database provides infectious disease surveillance data for the Member States in the European region. For hepatitis A, CISID presently includes reported annual cases and annual case rates (expressed as cases per 100,000 population) by country. This database is very powerful, but it lacks the age-specific hepatitis A information that is critical for monitoring changing HAV epidemiology over time. Furthermore, all reporting entities may not be using uniform case definitions that include laboratory confirmation, and that may limit the comparability and reliability of the statistics in this database. Even with these shortcomings in the CISID database, the creation of similar systems in other world regions would be highly beneficial to public health. Collecting both historical and current data from the smallest subnational geographic units possible would allow for epidemiological changes to be quickly detected and for policies to be updated in a timely manner. Such a database would also allow for the development of better predictive models based on socioeconomic indicators that are known to be strongly correlated with hepatitis A incidence [1]. Ideally, members of the hepatitis A research and practice community can work together to create and maintain an improved data sharing and data management system. In the meanwhile, mapmakers should be sure to provide information about their data sources and the limitations of the currently available data and estimates, in addition to following other good mapmaking practices such as making sure that each map has a clear title and a legend that identifies the nature of the data presented and the sources of the data (including their years of collection and references to the documents from which the data were compiled).

## Conclusions

Improved water and sanitation systems in many parts of the world have saved millions of children from severe diarrheal disease, and allowed those individuals to reach adulthood without having been exposed to the hepatitis A virus and developed immunity to it. This is the paradoxical nature of hepatitis A risk: the lower infection rate associated with development may be responsible for increasing morbidity and mortality from the disease. The growing population of susceptible adults worldwide makes the availability of current, accurate, and valid risk maps important for public health and individual wellbeing. The tools we propose here would help policymakers, health practitioners, and researchers to better understand and address the changing epidemiology of HAV infection and the emerging threat from hepatitis A disease.

Similar challenges are associated with the creation of maps for other complex and emerging infections, especially vaccine-preventable infections for which maps are used to disseminate vaccination recommendations. The solutions we propose for these hepatitis A maps would apply equally well to those other conditions.

## Methods

### Literature review

Five hepatitis A maps were selected for comparison based on a review of the maps presented in published articles indexed in PubMed, a review of the websites of major health organizations such as the World Health Organization (WHO) and the U.S. Centers for Disease Control and Prevention (CDC), image searches using major Internet search engines, and consultation with experts of travel medicine and hepatitis A epidemiology. Two of the five maps selected were produced by health agencies, one from the WHO's International Travel and Health book (2008) [23] and one from the CDC's Yellow Book (2008) [24] that focuses on travel health. The remaining three maps were from peer-reviewed journal articles [9,25,26]. The titles, data sources, target audience, definitions of risk, risk levels, and notes included with each map were examined.

### Mapping hepatitis A risk

Global hepatitis A risk maps for 1990 and 2005 were produced in consultation with the World Health Organization's Public Health Mapping and Geographic Information Systems (GIS) program. Age-specific seroprevalence estimates for 1990 and 2005 for each of the 21 Global Burden of Disease (GBD) world regions were derived from curves fit to pooled data collected between 1985 and 1994 and between 1995 and 2008, respectively, as reported elsewhere [9]. Risk is indicated by the age at midpoint of population immunity to HAV, which is defined as the age at which at least half of the population in that age group has anti-HAV IgG antibodies indicating past exposure to the virus. Each of the GBD world regions was categorized as a low, intermediate, high, or very high risk area based on its age at midpoint of population immunity (for Figures 1 and 3) or susceptibility (for Figure 2).

## Competing interests

The authors declare that they have no competing interests.

## Authors' contributions

All authors contributed to the conception and conduct of the analysis and to the writing and revision of the manuscript. All authors have reviewed and approved the final text.
